# Deciphering mechanisms of *bla*_NDM_ gene transmission between human and animals: a genomics study of bacterial isolates from various sources in China, 2015 to 2017

**DOI:** 10.2807/1560-7917.ES.2023.28.37.2200925

**Published:** 2023-09-14

**Authors:** Kaichao Chen, Miaomiao Xie, Ning Dong, Edward Wai Chi Chan, Rong Zhang, Sheng Chen

**Affiliations:** 1 Department of Infectious Diseases and Public Health, Jockey Club College of Veterinary Medicine and Life Sciences, City University of Hong Kong, Kowloon, Hong Kong Special Administrative Region, China; 2 These authors contributed equally to this work; 3 State Key Lab of Chemical Biology and Drug Discovery, Department of Applied Biology and Chemical Technology, The Hong Kong Polytechnic University, Hung Hom, China; 4 Department of Clinical Laboratory, Second Affiliated Hospital of Zhejiang University, School of Medicine, Zhejiang, Hangzhou, China

**Keywords:** *Escherichia coli*, *bla*_NDM_ gene, Shared ST types, ST10, ST167, transmission

## Abstract

**Background:**

In China, the *bla*_NDM_ gene has been recovered from human bacterial isolates since 2011. After 2014, detections of this gene in animal and food bacterial isolates have increasingly been reported.

**Aim:**

We aimed to understand how *bla*_NDM_-bearing bacteria could spread between humans, animals, and animal-derived food.

**Methods:**

A total of 288 non-duplicate *Escherichia coli* strains, including 130 *bla*_NDM_-carrying and 158 *bla*_NDM_-negative strains were collected from clinical (humans), food-producing animals (pigs) and food (retail pork) sources between 2015 and 2017. The strains were whole genome sequenced. Core-genome-multilocus-sequence-typing was conducted. To investigate if sequence types (STs) found in human, animal or food samples could have a prior origin in a clinical, animal or food-borne animal reservoir, discriminant analysis of principal components (DAPC) was used. Plasmids bearing *bla*_NDM_ were characterised.

**Results:**

The 130 *bla*_NDM_-carrying *E. coli* strains comprised a total of 60 STs, with ST167 (10/51), ST77 (6/33) and ST48 (6/46) being most prevalent in clinical, animal and food sources, respectively. Some ST10 and ST167 strains were respectively found among all three sources sampled, suggesting they might enable transfer of *bla*_NDM_ between sources. DAPC analysis indicated possible transmissions of ST167 from humans to animals and ST10 from animals to human. In 114 of 130 *bla*_NDM_-carrying isolates, *bla*_NDM_ was located on an IncX3 plasmid.

**Conclusion:**

This study in a Chinese context suggests that cross-species transmission of certain STs of *E. coli* harbouring *bla*_NDM_ on mobile elements, may facilitate the spread of carbapenem-resistant Enterobacteriaceae. Stringent monitoring of *bla*_NDM_-bearing *E. coli* in ecosystems is important.

**Key public health message**

**What did you want to address in this study?**

Treating infections caused by antimicrobial-resistant bacteria can be challenging. In China, bacteria bearing *bla*_NDM,_ a gene conferring antimicrobial resistance (AMR), have been reported in people already since 2011. After 2014, reports of detections of *bla*_NDM_ in bacteria from animals and food increased. We wished to understand how the *bla*_NDM_ gene might have spread between bacteria found in humans, animals, and food (retail meat) in China.

**What have we learnt from this study?**

We investigated the *bla*_NDM_ gene in *Escherichia coli* bacteria. Some types of *E. coli* occur more often in humans, others in animals. We detected two *E. coli* types (ST10 and ST167) each commonly in humans, animals, and food. This suggested that they could potentially act as vehicles for the transfer of *bla*_NDM_ between these three sources. Further analyses pointed to possible ST167 transmissions from human to animal and ST10 from animal to human.

**What are the implications of your findings for public health?**

This study provides novel understanding on how *bla*_NDM_ genes could potentially spread in the ecosystem in China. Intensified surveillance of ST10 and ST167 strains in animals and the food production chain may allow to find effective approaches to minimise the dissemination of *bla*_NDM_ in different environmental settings and the related threat to public health. This study emphasises the importance of studying AMR under a One Health framework.

## Introduction

Antimicrobial resistant bacteria pose an important threat to human and animal health. Globally, the dissemination of carbapenem-resistant *Enterobacteriaceae* (CRE) in particular has resulted in an increasing incidence of infections associated with high mortality [1,2]. In the European Union/European Economic Area (EU/EEA), the median numbers of infections and attributable deaths by CRE in 2015 were estimated to range from 2,619 and 141 for *Escherichia coli* to 61,892 and 4,155 by *Pseudomonas aeruginosa*, respectively [3].

Among several possible mechanisms conferring resistance in CRE, one can be the production of serine β-lactamases, including the oxacillinase (OXA) enzymes that were originally expressed by *Acinetobacter baumannii* and the *Klebsiella pneumoniae* carbapenemase (KPC) [2]. Resistance can also be due to the production of metallo-β-lactamases (MBLs), such as New Delhi metallo-β-lactamase (NDM) [2], and a key mechanism underlying the increasing prevalence of CRE strains is the acquisition of *bla*_NDM_ or *bla*_KPC_ genes [4].

While several variants of the *bla*_NDM_ gene (e.g. *bla*_NDM-1_ or *bla*_NDM-5_) exist [5], the *bla*_NDM_ gene product is generally defined as a type of Ambler class B MBL that exhibits high hydrolytic ability against almost all β-lactam antibiotic agents, including carbapenems, which are considered last resort antibiotics [6]. The *bla*_NDM_ gene was initially reported in 2009, as recovered from an *K. pneumoniae* sequence type (ST) 14 in a patient who had travelled to India [7]. Since, it has been detected in various parts of the world [8]. In China, a *bla*_NDM_-bearing strain was first reported in 2011 [9].

As does *bla*_KPC_, the *bla*_NDM_ gene can locate either on the chromosome or on plasmids [10]. Khan et al. demonstrated that the *bla*_NDM-1_ gene can be harboured by plasmids of IncFII, IncL/M, IncN, IncR and InHIB-M/FIB-M groups, with the IncFII type being the most prevalent [11]. All plasmids of these groups exhibit high efficiency of transmission mediated by various conjugation mechanisms [12-15].

Asymptomatic intestinal carriage of CRE in people has been reported as relatively common in some parts of China [16]. Currently, CRE are often detected in healthcare settings [17,18], where they are frequently recovered during faecal screening of patients in hospitals [19]. Strains of *bla*_NDM_-bearing bacteria are mainly recorded from patients with symptomatic infections [20]. While reports of *bla*_NDM_-carrying strains from both animal and food sources remained scarce until 2014 [21,22], accounts of especially *bla*_NDM_-carrying CRE strains appeared to thereafter increase [17,23], with detections in swine and cow farms [24,25]. In poultry, *Escherichia coli* containing the *bla*_NDM_-carrying IncX3 plasmids has also been observed [26]. Moreover, food products contaminated with CRE including pork, chicken and beef have been described [27,28].

This may suggest that *bla*_NDM_-bearing CRE strains might have become increasingly transmitted across species, also spreading to meat products. This study investigates *bla*_NDM_-bearing *E. coli* strains to understand their spread between humans, animals, and food.

## Methods

### Bacterial isolation

A total of 288 non-duplicate *E. coli* strains were recovered in China from clinical and food samples, as well as animal faeces. Among them, 130 clinical strains (from symptomatic human patients) were collected in a nationwide survey in 2015 [18], 66 animal faecal *E. coli* strains were recovered from two pig farms in 2017 in Huai’an and Taicang, Jiangsu Province and 92 food-borne strains from retail meat (pork) were isolated in Shenzhen from 2015 to 2017 [28]. Species identification was conducted by performing matrix-assisted laser desorption ionisation-time of flight mass spectrometry (MALDI-TOF MS). Susceptibility to antibiotics was tested according to Clinical and Laboratory Standards Institute (CLSI) guidelines [29], with the reference strain *E. coli* ATCC 25922 used for quality control. All bacterial strains were tested for the presence of *bla*_NDM_ gene by PCR assay as previously described [12].

### Whole genome sequencing and genomic analysis

Total DNA of the test strains was extracted by Pure-Link genomic DNA Mini kit (Invitrogen, US). Nextera XT DNA sample preparation kit (Illumina, San Diego, California, US) was used to construct DNA libraries. The samples were sequenced in an Illumina Hiseq X machine for 300 cycles (150 bp paired end). Trimmed and quality-filtered Illumina reads were acquired by using Trimmomatic v0.36 [30]. Bacterial genome sequences were achieved by using SPAdes version 3.12.1 [31] and CLC Genomics Workbench (CLC bio, Denmark). Species identification, multilocus sequence typing (MLST) and core-genome MLST (cgMLST) of the test isolates were confirmed based on the PubMLST database (https://pubmlst.org/) and SeqSphere + v3.4.0 (Ridom GmbH, Munster, Germany, http://www.ridom.de/seqsphere/), respectively. The assembled genomes were annotated by the Rapid Annotations using Subsystems (RAST) [32] and the National Center for Biotechnology Information (NCBI) Prokaryotic Genome Annotation Pipeline (PGAP). Whole-genome phylogenetic trees involving the reference strain were generated for the characterisation of *E. coli*. To detect antimicrobial resistance (AMR) genes, including *bla*_NDM_, and assess the distribution patterns of such genes in plasmids, draft genome mappings were produced by Basic Local Alignment Search Tool (BLAST; http://blast.ncbi.nlm.nih.gov/Blast), ResFinder [33], PlasmidFinder [34] and the CLC Genomics Workbench (CLC bio, Denmark). A minimum spanning tree (MST) was generated using SeqSphere + v3.4.0. 

### Phylogenetic analysis

Trimmed and quality-filtered assembly sequences acquired from the test *E. coli* strains were mapped to the *bla*_NDM_-bearing strain 40, which was a food-borne isolate recovered at the earliest date of the study (2015). Single-nt polymorphisms (SNPs) were performed by Snippy v3.1 with default parameters [35], using BWA-MEM v0.7.12 for short read mapping. Snippy produced two profiles, namely a core SNP alignment and a core-full SNP alignment. The core-full SNP alignment profile was used to infer the maximum likelihood (ML) phylogenies using Fasttree v2.1.10, with default settings [36]. The phylogenetic tree was visualised by iTOL version 3 [37]. MLST analysis, which included 80 known MLST-type strains and 16 new MLST-type isolates, was also performed for the purpose of clustering analysis [38].

### Source predictions for carbapenem-resistant strains by discriminant analysis of principal components

Discriminant analysis of principal components (DAPC) is a multivariate method designed to identify and describe clusters of genetically related individuals and to assign individuals to groups using the adegenet package implemented in the free software R (R Development Core Team; version 4.0.2) [39]. To set up a detectable DAPC model, we downloaded 18,230 metadata files, including the source and sampling site information for *E. coli*, from the NCBI website (https://www.ncbi.nlm.nih.gov/pathogens). In total, 5,902 E. coli isolates with metadata documenting their collection from clinical settings, food (pork) and animals (pigs) were subjected to screening. The strains were randomly selected using the Rand function in Excel 2020, 10 per cent of the strains were chosen from groups including > 1,000 database entries. Whole-genome sequencing data for 583 *E. coli* strains including 362, 120, and 101 recovered from clinical settings, food, and animals, respectively, were downloaded from the NCBI databases. The core-genome SNP profiles of each of the 583 *E. coli* isolates were generated using Parsnp, and an SNP matrix based on these profiles was generated using HarvestTools [40]. The SNP matrix of the 583 strains from the three origins (clinical, animal and food samples) was then used to construct the DAPC model as previously described [39]. Genome assembly of 50 isolates from each of clinical settings, food, and animals from the remaining 5,319 isolates were randomly selected to test the model. Finally, the constructed model was used to assess the degree of genetic relatedness of all CRE isolates collected from different sources in this study.

## Results

### Bacterial isolation

A total of 288 non-duplicated *E. coli* isolates were investigated. Of these, 130 were found to harbour the *bla*_NDM_ gene and exhibit the carbapenem resistance phenotype. Among these 130 isolates, 51, 46, and 33 strains were isolated from clinical specimens, animal food samples and animal faecal samples, respectively (Table 1). The *E. coli* isolates had been recovered at different times and geographical locations as shown in Supplementary Figure S1. The 51 *bla*_NDM_-positive clinical strains had been obtained in 2015 from infected symptomatic patients in hospitals across 14 Chinese provinces (representing ca two-fifth of the provincial-level administrative regions in the country). The food-borne strains had been collected from pork samples between 2015 and 2017 in Shenzhen, with *bla*_NDM_ detection rates appearing to increase each year; 0.3% (1/385) in 2015, 1% (5/570) in 2016 and 17% (40/236) in 2017 [28]. The animal-derived *bla*_NDM_-positive strains were from 2017 and isolated from two pig farms in Jiangsu Province, accounting for 17.1% (33/193) of the farm collection. Antimicrobial susceptibility tests of the 130 *bla*_NDM_-positive strains showed that these isolates exhibited very high resistance rates to most of the antibiotics tested (Table 1). A total of 30 AMR genes were identified in the 130 *bla*_NDM_-positive *E. coli* isolates, conferring resistance to nine classes of antimicrobial agents and this is illustrated in Supplementary Figure S2. The resistance phenotype in *E. coli* strains derived from three different sources was similar except for colistin resistance conferred by *mcr-1* gene, which exhibited a higher prevalence rate in *E. coli* strains recovered from food and animals (45.4%, 15/33 and 30.4%, 14/46, respectively) when compared with that of clinical isolates (7.8%, 4/51) (Table 1). Further details on analyses and findings regarding resistance are described in the Supplementary Materials.

**Table 1:** Characteristics of *bla*_NDM-_positive *Escherichia coli* isolates recovered from clinical, animal and food samples, including for each source the proportions of new sequence types and the resistance rates to antibiotics, China, 2015–2017 (n = 130 isolates)

Source	Number of isolates with *bla*_NDM_	Number of new STs/total STs^a^	Antibiotic^b^ resistance rate (%)												
			AMK^b^	AMP^b^	CRO^b^	CHL^b^	CIP^b^	CLS^b^	KAN^b^	MRP^b^	NAL^b^	STR^b^	TET^b^	SXT^b^	CAZ/AVB^b^
Clinical	51	2/24	17/51	49/51	51/51	26/51	33/51	4/51	32/51	51/51	49/51	35/51	42/51	49/51	51/51
Animal	33	2/14	8/33	32/33	33/33	26/33	14/33	15/33	18/33	33/33	26/33	23/33	31/33	33/33	29/33
Food	46	3/31	0/46	46/46	46/46	37/46	21/46	14/46	21/46	46/46	34/46	40/46	39/46	42/46	39/46

*E. coli*: *Escherichia coli*; STs: sequence types.

^a^ A total of 60 STs were identified, with nine STs being common to two or more sources: ST10 and ST167 were found among strains recovered from clinical, animal and food isolates; ST410 and ST155 were detected in both clinical and food-borne strains; ST48, ST641 and ST746 were observed in both clinical and animal isolates.

^b^ AMK: amikacin; AMP: ampicillin; CRO: ceftriaxone; CHL: chloramphenicol; CIP: ciprofloxacin; CLS: colistin; KAN: kanamycin; MRP: meropenem; NAL: nalidixic acid; STR: streptomycin; TET: tetracycline; SXT: trimethoprim/sulfamethoxazole; CAZ/AVB: ceftazidime−avibactam.

### Genetic characteristics of *bla*_NDM_-bearing *Escherichia coli* strains recovered from animals, clinical samples, and food products

**Figure 1:**
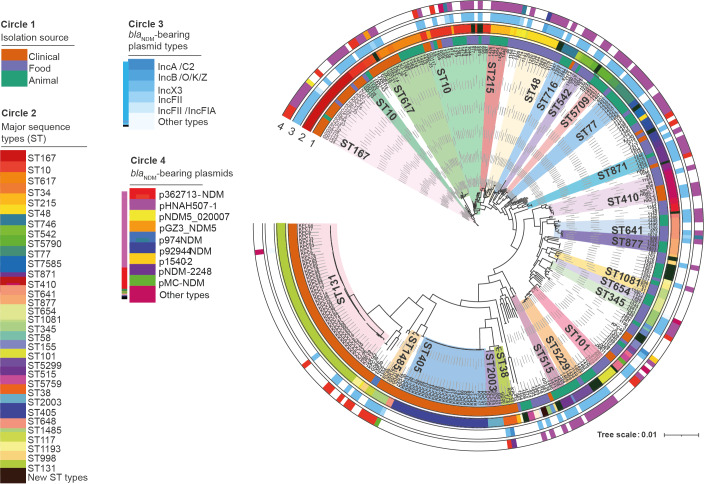
Phylogenetic tree of 130 *bla*_NDM_-bearing and 158 *bla*_NDM_ negative *Escherichia coli* isolates of different STs recovered from three different sources, China, 2015–2017 (n = 288 isolates)

MLST: multilocus sequence typing; ST: sequence type.

MLST and cgMLST revealed a total of 19 new and 80 known STs among the 288 *E. coli* strains. No obvious genetic differences between *bla*_NDM_-negative and positive strains of the same STs were observed (Figure 1). Among the 130 *bla*_NDM_-bearing *E. coli* strains, a total of 60 STs were detected, including 53 known STs and seven new types. The distribution pattern of STs according to the results of phylogenetic analysis (Figure 1) was distinctive among *E. coli* strains recovered from clinical, animal and food samples, as also depicted in Supplementary Figure S3.

Food sequences of 46 *bla*_NDM_ positive strains in the tree originated from animal products including pork (n=37), chicken (n = 7), shrimp (n = 1) and beef (n = 1). Circle 1 depicts strain collection source. Circle 2 denotes distribution of STs, with the different colours on this circle representing the main ST in each clade of the tree. Circle 3 in different shades of blue denotes distribution of plasmid types among *bla*_NDM_ positive strains. Circle 4 denotes the distribution of different *bla*_NDM_-bearing plasmids in this study. The maximum-likelihood tree, rooted with *bla*_NDM_-bearing strain 40 (Genbank accession number: GCA_025798185.1), was built based on the whole genome sequences of the isolates.

For the 51 *bla*_NDM_-carrying clinical *E. coli* isolates, ST167, ST131, and ST10 were the most common STs, accounting for 11, five, and five isolates, respectively; the predominant STs among the 33 animal *bla*_NDM_-carrying *E. coli* strains were ST77, ST215, and ST5299, which accounted for six, five, and four animal-borne *bla*_NDM_-carrying *E. coli* strains, respectively. Food isolates exhibited the highest level of ST diversity, in which a total of 34 STs were identified among the 46 strains, with ST48 being the most prevalent (6/46), followed by three STs (ST10, ST345 and ST87), each accounting for four of the 46 strains. Interestingly, most STs were source specific, except ST10 and ST167 which were found distributed among strains recovered from all three sources. Furthermore, ST410 and ST155 were detected in both clinical and food-borne *E. coli* strains; likewise, ST48, ST641 and ST746 were common among clinical and animal isolates (Supplementary Figure S3).

**Figure 2:**
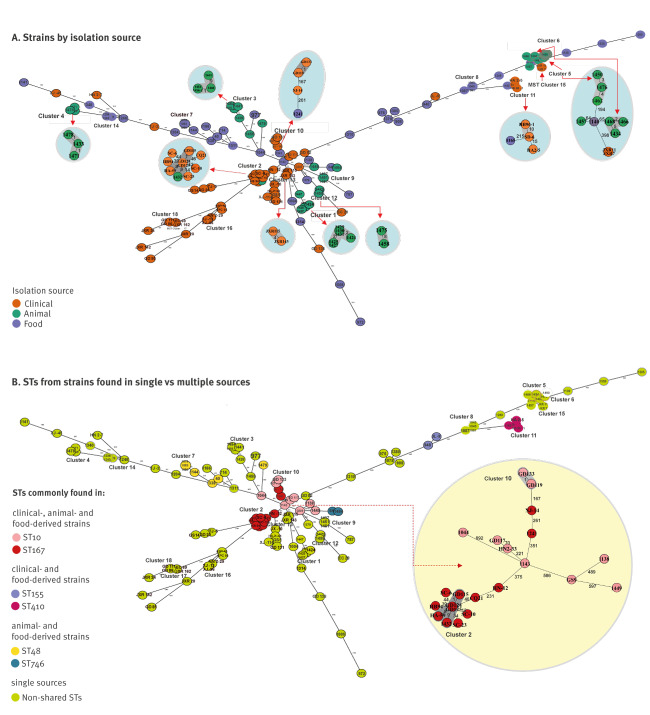
Minimum spanning tree based on cgMLST allelic profiles of *bla*_NDM_-positive isolates of different origins, China, 2015–2017 (n = 130 isolates)

To investigate the genetic relationship between these *E. coli* strains, cgMLST analysis, which involved core genome-wide-gene-by-gene comparison, was performed, with results showing a total of 18 distinct clusters (less than 10 allelic differences) on five branches of a minimum spanning tree (MST) (Figure 2). The three sources of bacteria are highlighted in the MST (Figure 2a). Clinical strains are mainly observed in one branch of the MST, whereas animal and food-borne strains do not exhibit specific distribution patterns. The distribution of the shared STs among strains from different sources is also depicted in MST, with ST10 and ST167 isolates appearing as being closely related (Figure 2b). Among the 13 ST167 strains, five were assigned to MST Cluster 2 and among the nine ST10 strains, two were assigned to Cluster 10. On the other hand, the 18 MST clusters identified by cgMLST indicated that *bla*_NDM_-bearing strains may have undergone horizontal dissemination in human, animals, and food, whereby the spread of *bla*_NDM_-bearing strains was common in animal (n = 8) and human (n = 7) clusters but less in food-borne (n = 2) and mixed clusters (n = 1).

cgMLST: core genome multilocus sequence typing; MST: minimum spanning tree; ST: sequence type.

Each circle depicts an allelic profile based on sequence analysis of 2,513 core-genes of *Escherichia coli*. Clusters of strains with closely related genotypes (≤ 10 alleles difference) are indicated by grey shades between the circles; some clusters are amplified for better visualisation with a blue background in panel A and a yellow background in panel B. Five main branches can be observed in MST, with clusters 5, 6, 8, 11 and 15 stemming from branch 1; clusters 3, 4, 7 and 14 stemming from branch 2; clusters 2, 16 and 18 stemming from branch 3; clusters 1 and 13 stem from branch 4; clusters 9 and 12 stemming from branch 5.

A. Colours of circles denote different collection sources. Red is used to highlight bacteria recovered from animals. Green and purple depict clinical and food-borne *bla*_NDM_ pathogens, respectively.

B. Colours of different circles represent the distribution of STs of bacteria recoverable from two or more sources, the light pink and red circles represent strains of ST10 and ST167, respectively. Fuchsia, lavender blue, teal and yellow depict strains of ST410, ST155, ST746 and ST48, respectively. Light green denotes the STs of strains recovered from a single source.

### Source predictions for carbapenem-resistant *Escherichia coli* strains by discriminant analysis of principal components model

**Figure 3:**
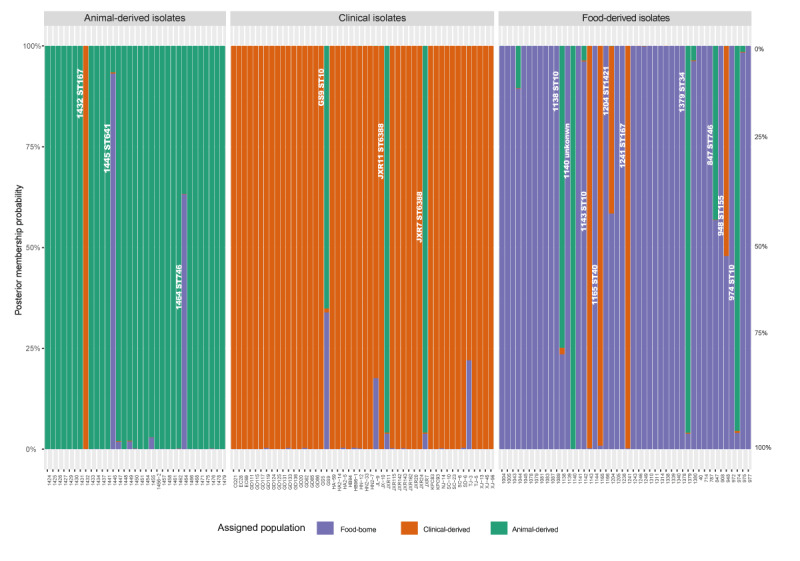
Posterior membership probability of 130 *bla*_NDM_ positive bacteria derived from three origins, China, 2015–2017 (n = 130 isolates)

Possible mechanisms of spread or transmission of *bla*_NDM_-bearing plasmids among *E. coli* strains in different sources were investigated. Overall, the first 200 principal components and 51 discriminant functions were retained to construct the training model. DAPC was performed on the 130 *bla*_NDM_-positive strains to further examine the relationship between the three groups of isolates; analysis of similarity was performed to test if the differences between the test strains were considerable. DAPC analysis showed these bacteria belonged to three distinct clusters, which only partially overlap with each other. Strains from three categories in different sources (human, animal and food) were properly differentiated in the training model as indicated in Supplementary Figure S4. The prediction accuracy rate of the 130 selected *E. coli* for each source ranged from 32.8% to 82.4%. Posterior membership probability was achieved among them, and most of the test strains were proven to match their own collection source; however, as shown in Supplementary Table S1, nearly 12.3% (16/130) of the strains exhibited high possibility of having been transferred from other sources (Figure 3). Food-borne isolates were apparently derived from exogenous sources, with 10 strains being assigned to clinical and animal origins, respectively (four of them exhibited 99% eventuality). Three clinical strains were assigned to animals (ST10, n = 1; ST6338, n = 2) and three animal strains were assigned to clinical (n = 1, ST167) and food sources (n = 2, ST641, ST746). The detection of a clinically-originating ST167 among isolates from animals in this study (isolate 1432, also visible within cluster 2 in Figure 2 together with clinical isolate GD124) further supported the possibility of transmission of *bla*_NDM_-carrying ST167 *E. coli* from human to animals; the detection of animal-originating ST10 and ST6338 isolates among the clinical isolates from the current study suggested the transmission of *bla*_NDM_-bearing *E. coli* from animal back to human (Figure 3, Table S1). In food samples collected in China, the higher diversity of *E. coli* isolates likely originating from an animal and clinical source was consistent with its role as a vehicle for dissemination.

ID: identifier.

The three separate parts depict the respective collection source of *bla*_NDM_ positive isolates involved in this study. ID of test strains are shown at the bottom. Green indicates strains recovered from animal farms, orange, strains from clinical isolates and purple, strains from food samples. The scales on each side depict the posterior membership probability, depending on whether the bars are based on the top or from the bottom of the graph.

### Genetic characterisation of *bla*_NDM_-bearing *Escherichia coli* strains from three origins

Nucleotide BLAST (BLASTN) showed that all *bla*_NDM_-bearing genetic elements from clinical, food and animal-derived strains in China were in the form of plasmids. The variants of *bla*_NDM_ in the 130 strains included *bla*_NDM-5_ (n = 86), *bla*_NDM-1_ (n = 37), *bla*_NDM-7_ (n = 2), *bla*_NDM-9_ (n = 2), truncated Δ*bla*_NDM_ (n = 2) and *bla*_NDM-4_ (n = 1), among which the size of the 128 *bla*_NDM_-containing contigs ranged from 1,407 bp to 50,056 bp. The plasmids could be categorised into three clusters, each with a main type of genetic environment, namely Type a (n = 6), Type b (n = 118) and Type c (n = 4); this is depicted in Supplementary Figure S5. 

Type a cluster contained *int*-*dfrA12*-*hp*-*aadA2*-*sul1* and ΔIS*Aba125*-*bla*_NDM_-*ble*-*trpF* and distributed in clinical samples (n = 3) and pork samples (n = 3). Plasmid screening for these six strains showed that Type a sequence fragments with 4,681 and 10,390 bp lengths from clinical *E. coli* strains aligned well with two IncC type plasmids, namely p1540–2 (GenBank accession number: CP019053) and pNDM-2248 (GenBank accession number: MH844629), respectively. On the other hand, only one IncB/O/K/Z type plasmid p92944-NDM (GenBank accession number: MG838206) could be matched to a Type a fragment derived from food-borne *E. coli* strains. Additionally, there were three homologous contigs belonging to Type a, which were all 5,662 bp in length. Two of these, together with their backbone, which originated from food isolates mapped well to one IncHI2 type plasmid pJH51–1 (GenBank accession number: CP095455). The remaining one, with a clinical source, did not match any plasmid in the GenBank database (Table 2) as can be seen in Supplementary Figures S6, S7a and S7b).

**Table 2:** Prevalence and genetic characteristics of plasmids that harboured *bla*_NDM_ determinants in *Escherichia coli* strains, China, 2015–2017 (n = 125 isolates)^a^

Plasmid number	Reference	Size (bp)	Number of CDS in the plasmid	%GC	Plasmid types	*bla*_NDM_ (number of isolates)	Number of isolates	Number of new/total STs	Origin (number of isolates)	Accession number
1	pHNAH507–1	46,161	59	46.7	IncX3	NDM-1 (8), NDM-5 (78), NDM-7 (2), NDM-9 (1)	89	6/36	Animal (33), Clinical (18), Food (38)	MH286945
2	p362713-NDM	54,036	74	49.0	IncX3	NDM-1 (23), NDM-4 (1), NDM-5 (1)	25	1/14	Clinical (24), Food (1)	MH909347
3	pNDM-2248	182,653	242	52.0	IncC	NDM-1 (1)	1	0/1	Clinical (1)	MH844629
4	p1540–2	155,802	203	52.6	IncC	NDM-1 (1)	1	0/1	Clinical (1)	CP019053
5	p92944-NDM	118,644	160	54.6	IncB/O/K/Z	NDM-9 (1)	1	0/1	Food (1)	MG838206
6	p974-NDM	74,080	85	52.0	IncFII	NDM-5 (3)	3	0/3	Food (3)	MG825370
7	pGZ3_NDM5,	91,451	113	52.8	IncFII	NDM-5 (1)	1	0/1	Clinical (1)	CP017981
8	pMC-NDM	87,619	91	52.4	IncFII	NDM-1 (1)	1	0/1	Clinical (1)	HG003695
9	pNDM5_020007	144,225	187	53.2	IncFII/FIA	NDM-5 (1)	1	0/1	Clinical (1)	CP025626
10	pJH51–1	246,733	264	46.5	IncHI2	NDM-1(1), NDM-5(1)	2	0/2	Food (2)	CP095455

CDS: coding sequences; NDM: New Delhi metallo-β-lactamase; ST: sequence type; %GC: percentage of guanine and cytosine residues contained in the plasmid sequence.

^a^ Only 125 of the total 130 isolates in this study are presented in the table because for two strains, the obtained *bla*_NDM_ gene sequences were truncated and for three strains, the contigs containing *bla*_NDM-1_ genes did not match plasmids in the GenBank database.

The core genetic structure of the Type b contigs was *bla*_NDM_-*ble*-*trpF-dsbC*-*cutA*, surrounded by several insertion sequences (IS) including IS*5*, IS*Aba125*, IS*3100* and/or TnAs3. The Type b genomic sequences involved the contigs of 118 isolates, including those recovered from clinical samples (n = 46), animals (n = 29) and food products (n = 43). The complete *bla*_NDM_-bearing plasmids could be obtained from 25 *E. coli* isolates. These 25 plasmids all belonged to IncX3 plasmids with 14 aligning well to pHNAH507–1 (GenBank accession number: MH286945) and 11 aligning to p362713-NDM (GenBank accession number: MH909347), both of which shared the same core structures (Supplementary Figure S5**,** Table 2); this is illustrated in Supplementary Figure S8. Among the remaining 93 Type b contigs, 71 aligned well to the IncX3 plasmid pHNAH507–1 (ca 46 kb), 14 aligned well with an incX3 type plasmid p362713-NDM (ca 54kb) (Table 2**)**, which is also shown in Supplementary Figure S9, and eight could be aligned to a total of five different plasmids of three plasmid replicon types (IncFII (n = 5), IncFII/IncFIA (n = 1) and IncHI2 (n = 2)) (Table 2); additional depictions can be found in Supplementary Figures S10 and S11. Detail of these data can be found from the Supplementary Materials.

Type c contigs were all collected from animal-borne strains (n = 4). While the core genetic structure of *bla*_NDM_ elements in Type b and c fragments were similar, in Type c fragments the flanking mobile elements IS*3100* were replaced by a *fepE* gene, which encodes an inner membrane transport protein. Analysis of the backbone of plasmid sequences in Type c contigs showed that they also belonged to the IncX3 type (Supplementary Figure S5). Taken together, our data suggested that the transmission of *bla*_NDM_ in these isolates from different sources was mainly mediated by IncX3 plasmids with similar core structures.

## Discussion

The continuous appearance of new CRE strains limiting therapeutic choices poses challenges to treatment of multidrug-resistant infections in humans and animals. In this respect, CRE carrying the *bla*_NDM_ gene constitute a global health threat [9]. During the first few years following initial publications of *bla*_NDM_-bearing CRE strains, studies were mainly focused on humans, possibly because drugs selecting such strains, like carbapenems, might have not been considered used in animal husbandry [21,41]. Since the mid-2010s however, reports of *bla*_NDM_-bearing CRE strains recovered from animal and animal-derived food products seem to have increased [27,28,42].

While the reasons behind this are not clear, one potential explanation could be an increased spread of *bla*_NDM_-bearing CRE between humans, animals, and food. Should human-to-animal transmission occur, contamination of the environment by clinical waste and human faecal particles in some settings could be permissive to spread [43]. This would challenge efforts to control *bla*_NDM_-bearing CREs, since animals are excellent incubators of AMR gene-bearing organisms [44]. Moreover, for bacteria with *bla*_NDM_ on plasmids, transmission might be accelerated under antibiotic pressure in some places, such as for example in pig farms in China, where antibiotics such as amoxicillin, ceftiofur, tilmicosin, florfenicol, doxycycline and others are commonly used to prevent secondary infections [45].

In this study, we performed an in-depth genetic analysis on *bla*_NDM_-bearing and *bla*_NDM_-negative *E. coli* strains isolated from clinical, animal, and food sources. Phylogenetic analysis indicated that clinical *bla*_NDM_-bearing strains were genetically distinct from strains from animals and foods, which were mixed together, suggesting that transmission from people to animals is not a common event. 

Among *bla*_NDM_-bearing strains isolated in our study, certain STs nonetheless appeared to occur in more than one source. ST410 and ST155 were each detected in both clinical and food-borne strains; ST48, ST641 and ST746, were each found in clinical and animal isolates. Furthermore, isolates of ST10 and ST167 were recovered from all three clinical, animal, and food sources. In agreement with this result, ST167 *E. coli* has previously been reported as isolated commonly from humans and occasionally from animals [46-48]. Another study in 2022 showed that *bla*_NDM_-positive *E. coli* isolates of ST167 and ST10 belonged to two of four STs, which were shared between humans and animals, with 85.9% of ST167 and 62.1% of ST10 isolates respectively collected from humans and swine [49].

To further understand the possible spread of *bla*_NDM_-bearing *E. coli* between humans, animals and food, we then focused on searching strains of certain STs that had been sampled in either one of the three sources yet might have had an origin in another of these sources. Using DAPC analysis, we identified a few strains matching these criteria (Supplementary Table S1), which might possibly suggest their capacity to serve as a vehicle for the *bla*_NDM_ gene between the sources. These strains notably included two of ST167 (1241 and 1432) and four of ST10 (974, 1138, 1143 and GS9). 

Concerning ST167, cgMLST analysis of strains in the current study identified a *bla*_NDM_-bearing isolate from food (1241), which located near a cluster of clinical isolates in a MST (close to cluster 10). This food isolate was assigned by DAPC to a clinical origin, suggesting that ST167 strains with *bla*_NDM_ may be able transfer from patients to food. More importantly, cgMLST with MST showed that some ST167 strains which had been respectively sampled from a patient (GD124) and an animal (1432) clustered together (cluster 2) with an allelic difference of nine. The ST167 strain (1432) in MST cluster 2, which had been retrieved from an animal, was assigned by DAPC to a clinical source, providing some support for the transmission potential of ST167 between humans and animals.

For ST10, DAPC analysis suggested that strains of clinical origin might have transferred to food products, and the same was found for ST410. Moreover, ST10 and ST6388 strains from animals seemed to be transmittable to humans. The DAPC also assigned some STs found in food such as ST641 and ST746 to animals.

Among isolates collected in our study, most *bla*_NDM_-bearing plasmids found belonged to the IncX3 type. As this type appears to be common in animal and human bacteria [50], the various lines of evidence presented in this report, support a model whereby certain STs can serve as a vehicle for *bla*_NDM_ between species, followed by spread within the species via horizontal transfer. These *bla*_NDM_-bearing *E. coli* could be further transferred to food products and hypothetically back to humans as illustrated in Supplementary Figure S13. Nevertheless, direct transmission of food isolates back to human was not demonstrated in this work, but this might be due to the limited number of samples that we had.

Our study has some limitations. The role of bacterial species such as *Klebsiella pneumoniae* on the transmission of *bla*_NDM_-carrying IncX3 plasmids between humans and animals [51] was not investigated. This work reflected on the transmission of *E. coli* between patients and animals in a Chinese context; not all regions of China were sampled, and the number of isolates investigated was relatively small. Results might thus not be generalisable to the whole country. Furthermore, since the bacteria and plasmids involved in the spread of *bla*_NDM_ in different continents are very different [52], the findings may be not applicable to other parts of the world.

### Conclusion

Our study depicted possible mechanisms underlying the transmission of an important AMR gene in human and animals and provides novel understanding of *bla*_NDM_ gene spread in the ecosystem in China. Stringent monitoring of *bla*_NDM_-bearing *E. coli* of certain STs in farm animals and the food production chain may be an effective approach to find ways to minimise the dissemination of these multidrug-resistant strains in different environmental settings and their threat to public health.

## Supplementary Data

5671000 Supplementary Materials
