# Supplementary material for: Deciphering mechanisms of blaNDM gene transmission between human and animals: a genomics study of bacterial isolates from various sources in China, 2015 to 2017
Source: Euro Surveill. 2023 Sep 14;28(37):2200925. doi: 10.2807/1560-7917.ES.2023.28.37.2200925 (PMC10687984; doi:10.2807/1560-7917.ES.2023.28.37.2200925)
Supplement: Supplementary Materials [file 2200925_SupplementaryMaterials.pdf]

Supplementary materials

Deciphering mechanisms of *bla*$_{NDM}$ gene transmission between human and animals: a genomics study of bacterial isolates from various sources in China, 2015 to 2017

This supplementary material is hosted by Eurosurveillance as supporting information alongside the article [Deciphering mechanisms of *bla*$_{NDM}$ gene transmission between human and animals: a genomics study of bacterial isolates from various sources in China, 2015 to 2017] on behalf of the authors who remain responsible for the accuracy and appropriateness of the content. The same standards for ethics, copyright, attributions and permissions as for the article apply. Eurosurveillance is not responsible for the maintenance of any links or email addresses provided therein.

Supplementary results

Antimicrobial susceptibility of *bla*$_{NDM}$ positive strains.

Among the 288 *E. coli* isolates tested, 130 were found to harbor the *bla*$_{NDM}$ gene and exhibit the carbapenem resistance phenotype, among which 51, 46, and 33 strains were isolated from clinical specimens, food samples and animal fecal samples, respectively. Antimicrobial susceptibility tests showed that these isolates exhibited resistance to most of the antibiotics tested, with a resistance rate of 100% to meropenem and ceftriaxone. The rate of resistance of strains recovered from animal, clinical and food-borne samples to other antimicrobial agents were as follows: 96.9%, 96.0% and 100% to ampicillin, 93.9%, 82.3% and 84.7% to tetracycline, 100%, 96% and 91.3% to trimethoprim/sulfamethoxazole, and 87.9%, 100% and 84.8% to ceftazidime-avibactam, respectively. In addition, clinical isolates exhibited a slightly higher rate of resistance to ciprofloxacin, kanamycin and nalidixic acid than those derived from animals and foods, with the rate of resistance of clinical, animal, and food-borne isolates to ciprofloxacin being 64.7%, 42.4% and 45.6%. For kanamycin and nalidixic acid, the rate of resistance in clinical, animal, and food-borne isolates was 62.7%, 54.5% and 45.6%, and 96%, 78.7% and 73.9%, respectively. In contrast, clinical isolates displayed a lower rate of resistance to colistin and chloramphenicol (7.14% and 50.9%) when compared to that in animal and food-borne bacteria (45.4% and 78.7%, and 30.4% and 80.4%), respectively. Interestingly, bacteria derived from food and animal samples exhibited similar resistance rates against most of the antimicrobial agents, except for amikacin and streptomycin, for which the amikacin resistance rates in animal and food-borne bacteria were 24.2% and 0% and resistance rates of streptomycin in animal and food-borne bacteria were 69.7% and 86.9%, respectively.

Supplementary Figure S1. ***Bla*$_{NDM}$-positive *E. coli* isolates collection metadata.**

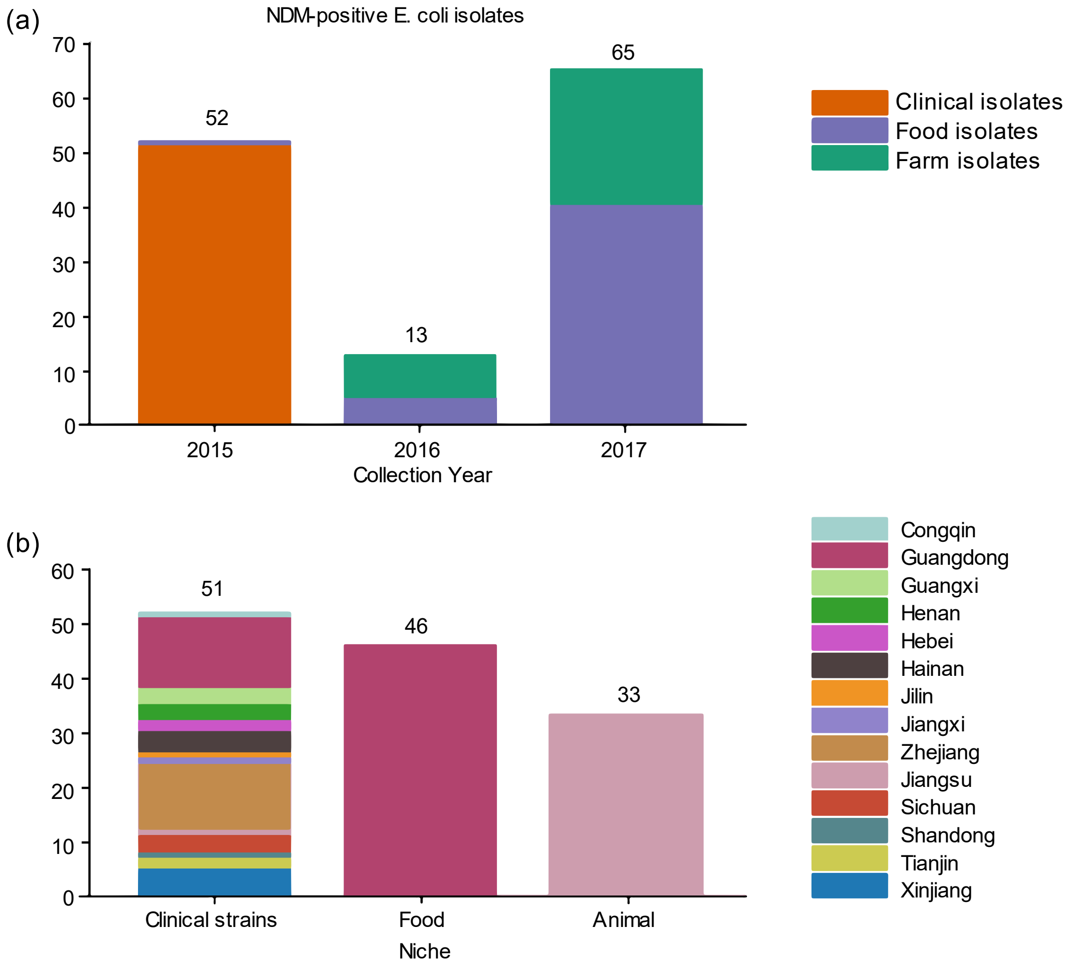

Summary of metadata of 130 *bla*$_{NDM}$-positive *E. coli* strains stratified by source. (a) collection year (2015-2017). (b) Niche.

Characterization of antimicrobial resistance gene profiles in *bla*$_{NDM}$-producing *E. coli* strains

A total of 30 types of resistance genes were detected among the 130 *bla*$_{NDM}$-producing *E. coli* strains, conferring resistance to nine classes of antimicrobial agents **(Fig S2)**. Ten of these genes, including *sul*, *bla*$_{NDM}$, *dfrA*, *tet*, *aadA*, *bla*$_{TEM}$, *strAB*, *floR*, *mph* and *aac*, conferred resistance to six classes of antibiotics, namely β-lactams, aminoglycosides, chloramphenicol, macrolides, sulfonamides and tetracyclines. These genes were present in more than 50% of the *bla*$_{NDM}$-producing *E. coli* strains. The distribution of AMRs in *E. coli* strains derived from

three different sources was similar except for the *mcr-1* gene, which exhibited higher prevalence rate in *E. coli* strains recovered from food and animals (48% and 30%, respectively) when compared to clinical isolates (7%) (**Fig S2**), inferring that the food and animal-borne *E. coli* strains often simultaneously harbored the *bla*$_{NDM}$ and *mcr-1* genes.

Supplementary Figure S2. Heatmap of 130 *bla*$_{NDM}$ positive *E. coli* isolates in three different sectors.

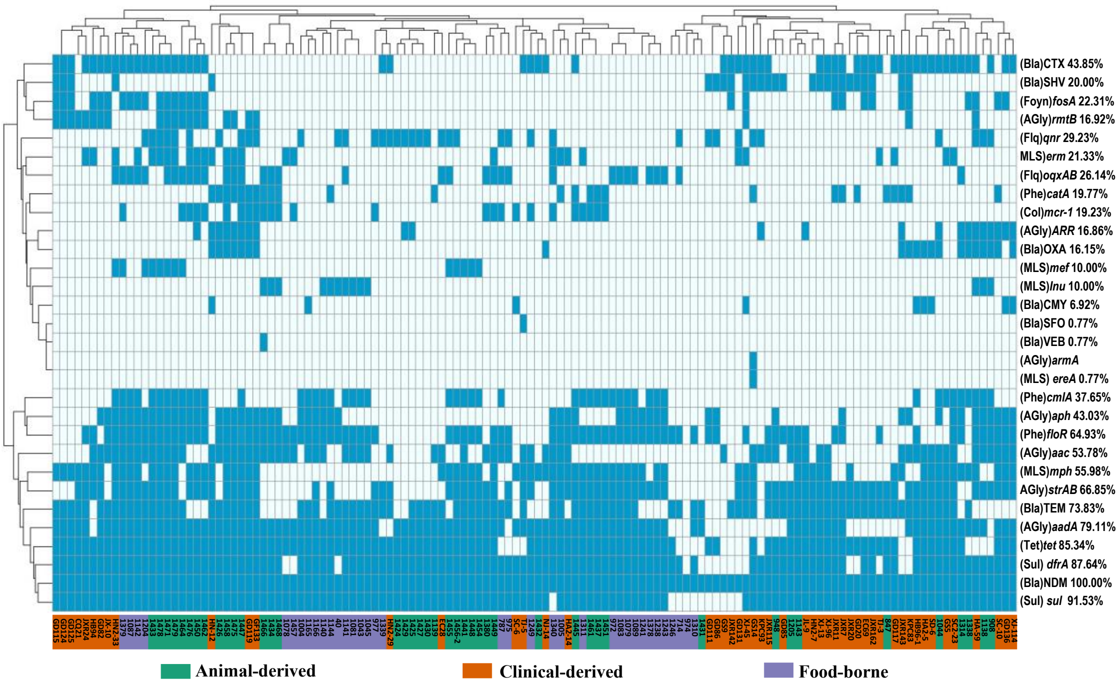

E. coli isolates are clustered using a maximum likelihood tree. Turquoise and white in the heatmap depict the presence and absence of AMRs in testing strains. The right side denotes the category of AMRs, with the corresponding detection rate. Green, orange, and purple indicate the origin of bacteria, consistent with the legend in the upper corner.

Supplementary Figure S3. Distribution of ST types of 130 blaNDM positive strains in animal, clinical and food E. coli isolates.

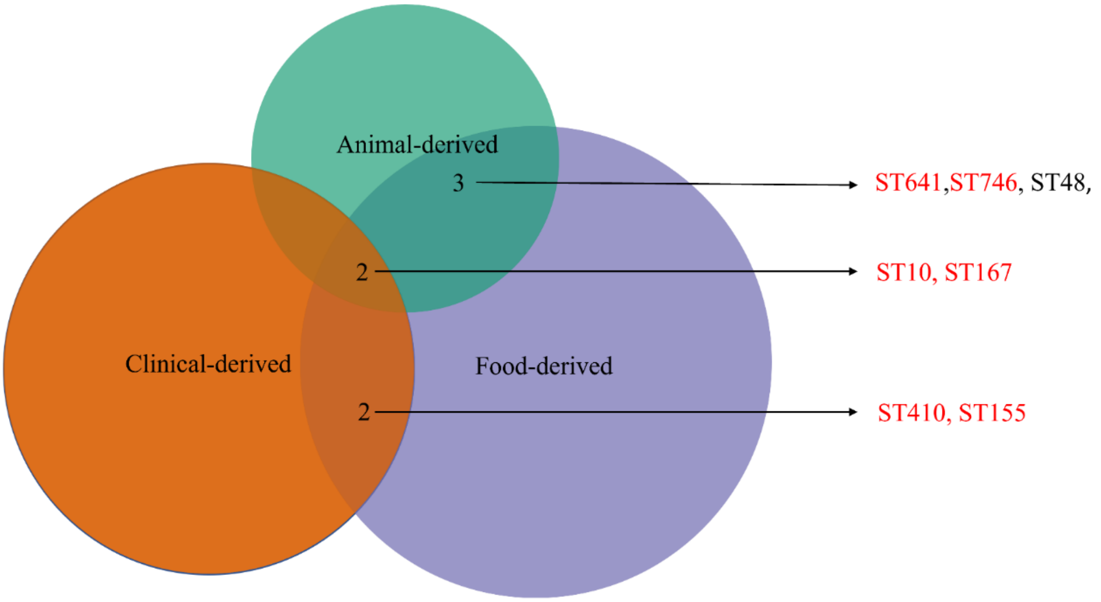

The arrows refer to shared ST type E. coli strains among three sectors, and red color precents the ST types that have undergone host transformation by DAPC analysis.

Supplementary Figure S4. Principal component analysis of 130 *bla*$_{NDM}$ positive strains in three different collection sources.

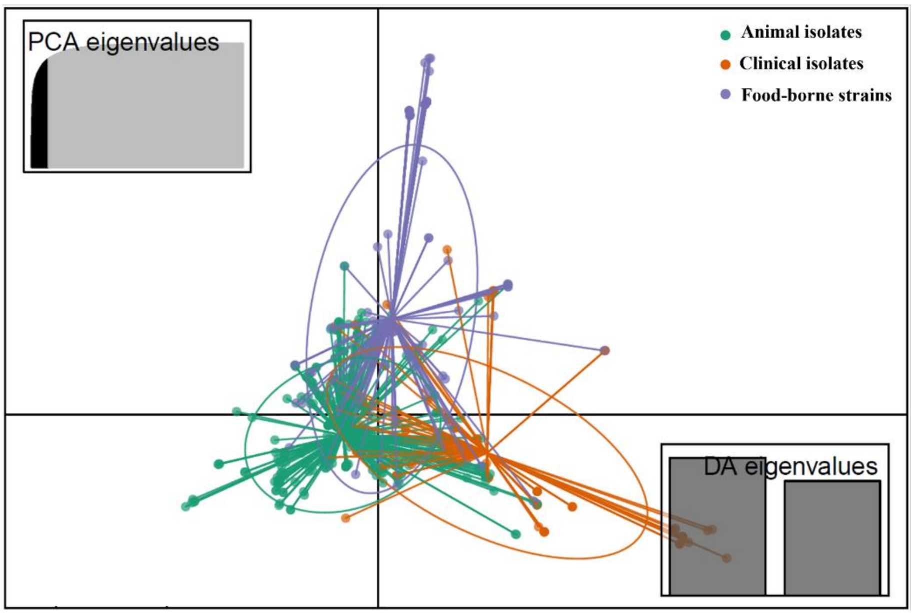

Principal component analysis displayed clear separation of three groups isolates, and each point precent corresponding test bacteria. Green, strains that collected from animal farms, Orange, bacteria that isolated from clinical specimen. Purple, isolates that recovered from food samples.

Supplementary Table S1. Characteristics of *E. coli* isolates with different assigned origins from origins of isolation.

Isolate Origin	ID	Assigned origin	confidence probability	ST types	*bla* NDM types	Plasmid types	Accession no.
Food	847	Animal	43.13%	746	NDM-7	IncX3	MH286945
Food	948	Clinical	52.11%	155	NDM-1	IncX3	MH909347
Food	974	Animal	95.47%	10	NDM-5	IncFII	MG825370
Food	1138	Animal	74.88%	10	NDM-5	Unknown	Unknown
Food	1140	Animal	99.93%	-	NDM-1	IncX3	MH286945
Food	1143	Clinical	99.79%	10	NDM-5	IncX3	MH286945
Food	1165	Clinical	99.15%	410	NDM-5	IncX3	MH286945
Food	1204	Clinical	41.54%	1421	NDM-5	IncX3	MH286945
Food	1241	Clinical	99.83%	167	NDM-5	IncX3	MH286945
Food	1379	Animal	95.91%	34	NDM-5	IncX3	MH286945
Animal	1432	Clinical	99.99%	167	NDM-1	IncX3	MH286945
Animal	1445	Food	93.11%	641	NDM-5	IncX3	MH286945
Animal	1464	Food	63.20%	746	NDM-5	IncX3	MH286945
Clinical	GS9	Animal	65.11%	10	NDM-1	IncX3	MH909347
Clinical	JXR11	Animal	95.89%	6388	NDM-1	IncX3	MH909347
Clinical	JXR7	Animal	95.89%	6388	NDM-1	IncX3	MH909347

Genetic characterization of *bla*$_{NDM}$-bearing *E. coli* strains from three origins

BLASTN showed that all *bla*$_{NDM}$ -bearing genetic elements were in the form of plasmids. The variants of *bla*$_{NDM}$ in the 130 strains included *bla*$_{NDM-5}$ (n=86), *bla*$_{NDM-1}$ (n=37), *bla*$_{NDM-7}$ (n=2), *bla*$_{NDM-9}$ (n=2), truncated Δ*bla*$_{NDM}$ (n=2) and *bla*$_{NDM-4}$ (n=1), among which the size of the 128 *bla*$_{NDM}$-containing contigs ranged from 1,407 bp to 50,056 bp, and can be categorized into three main types of genetic environment, namely type a (n=6), type b (n=118) and type c (n=4) **(Fig S5)**. Type (a) involved strains that were obtained from clinical samples (n=3) and pork purchased from supermarkets (n=3). The complete fragment of type (a) was found to be 10,390 base pair (bp) in size and comprise 13 predicted coding sequences (CDs). A class I integron with multiple antibiotic resistance genes (ARGs) are located downstream of the insertion sequence IS*CR1*. BLASTN showed that the genetic environment of the resistance cluster contained *int-dfrA12-hp-aadA2-sul1* and ΔIS*Aba125-bla*$_{NDM}$*-ble-trpF*, which encoded resistance to antimicrobial agents such as aminoglycosides, meropenem, sulfonamides and trimethoprim. In addition, plasmid screening was performed on these six strains, with results showing that contigs that contain the Type (a) fragment of clinical *E. coli* strains can be aligned well with two IncC type plasmids, namely pNDM-2248 (Accession no. MH844629), and p1540-2(Accession no. CP019053), yet only one IncB/O/K/Z type plasmid p92944-NDM (Accession no, MG838206) could be matched for type (a) fragment derived from foodborne *E. coli* strains, Additionally, three homologous contigs belonging type a were all 5,662 in length, two of which together with their backbone can be mapped well to one IncHI2 type plasmid pJH51–1 (accession number, CP095455), the rest one did not match plasmids in the GenBank database. (Table 2, Fig S6, S7a and S7b).

Supplementary Figure S5. Genetic environment of the blaNDM profile of 128 E. coli strains.

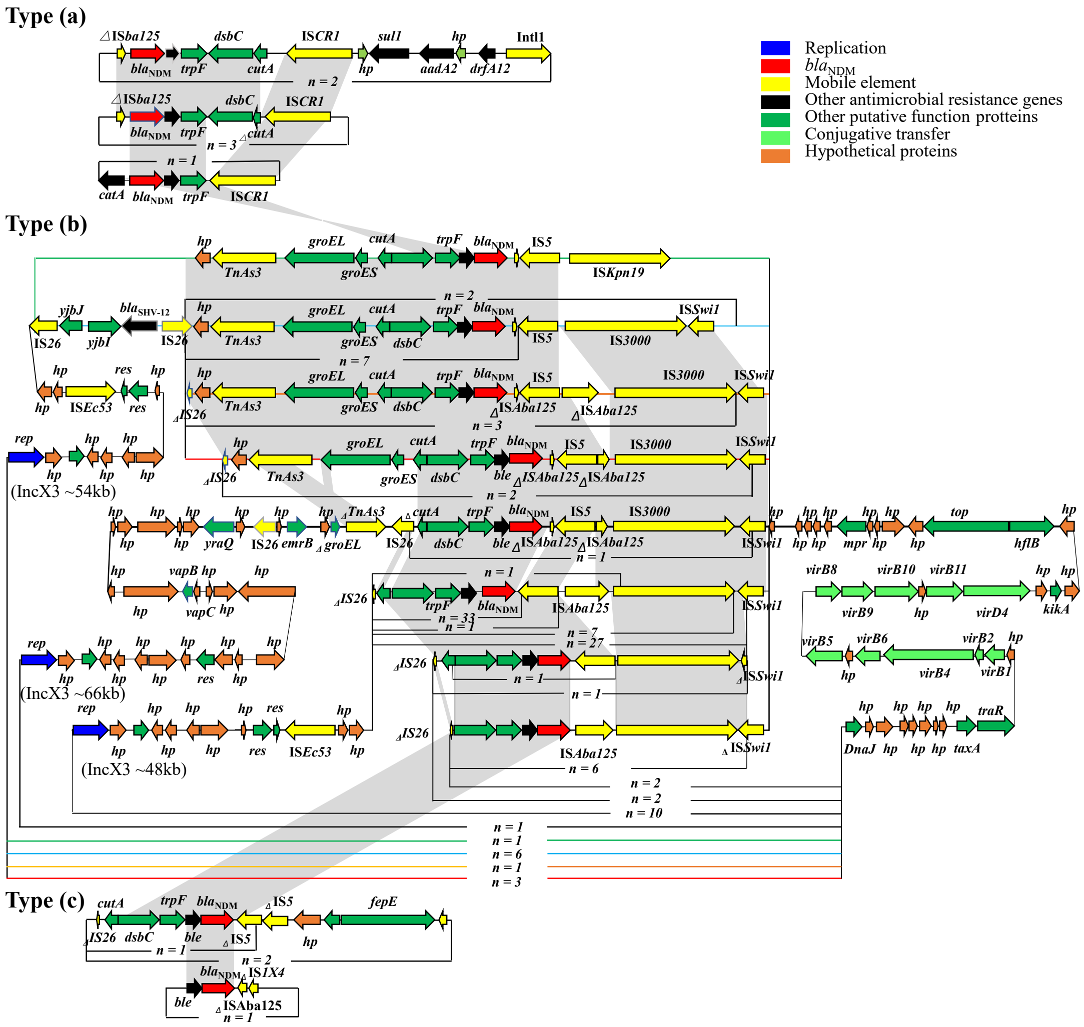

Horizontal arrows indicate the location, size, and orientation of predicted coding sequences (CDS), with different functions depicted by specified colors. Red, blaNDM gene; yellow, genes that encode insertion elements and integrases. Green, genes that encode the transfer protein. Blue, black, and orange, replication, other antimicrobial resistance and hypothetical protein-coding genes, respectively.

Supplementary Figure S6. Alignment of IncC plasmids pNDM-2248 and p1540-2 (NCBI database accession number MH844629 and CP019053, respectively) with its best BLAST hit in this study.

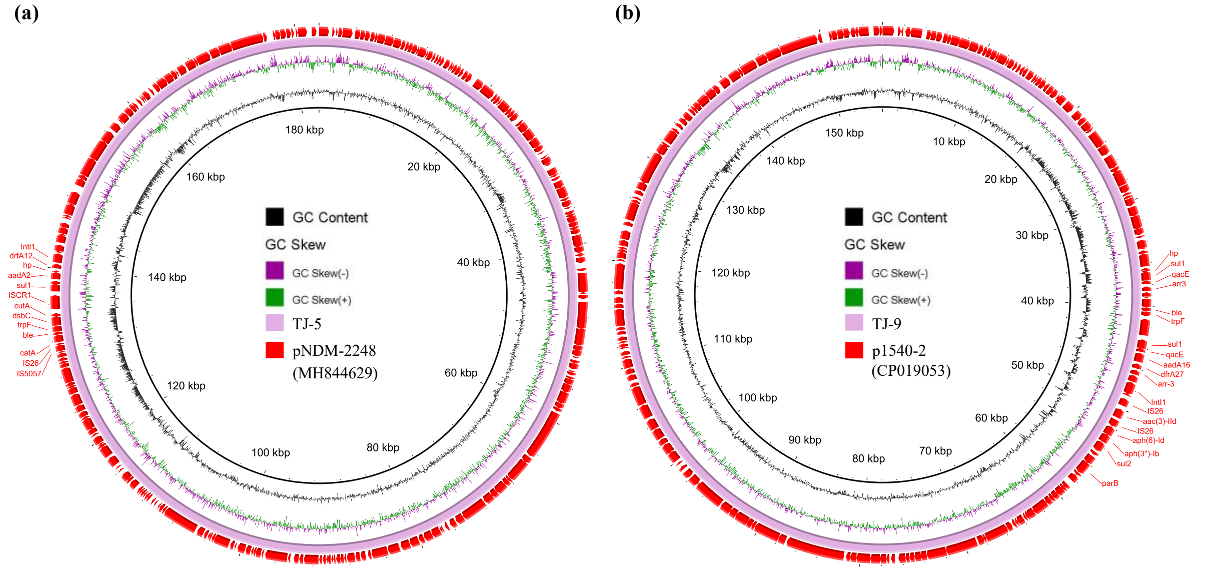

(a) Clinical strain TJ-5 was found to contain pNDM-2248-like plasmid, which was used as reference (the outmost circle); (b) Clinical strain TJ-9 was found to harbor p1540-2 -like plasmid, key genetic loci of integrons were labeled. Plasmid sequences in this study were generated from the Illumina sequencing data.

Supplementary Figure S7. Structure alignment of one IncB/O/K/Z plasmid p92944-NDM pJH51-1 (NCBI database accession number HG003695 and CP095455, respectively) (Accession no, MG838206) with its best BLAST hit in this study.

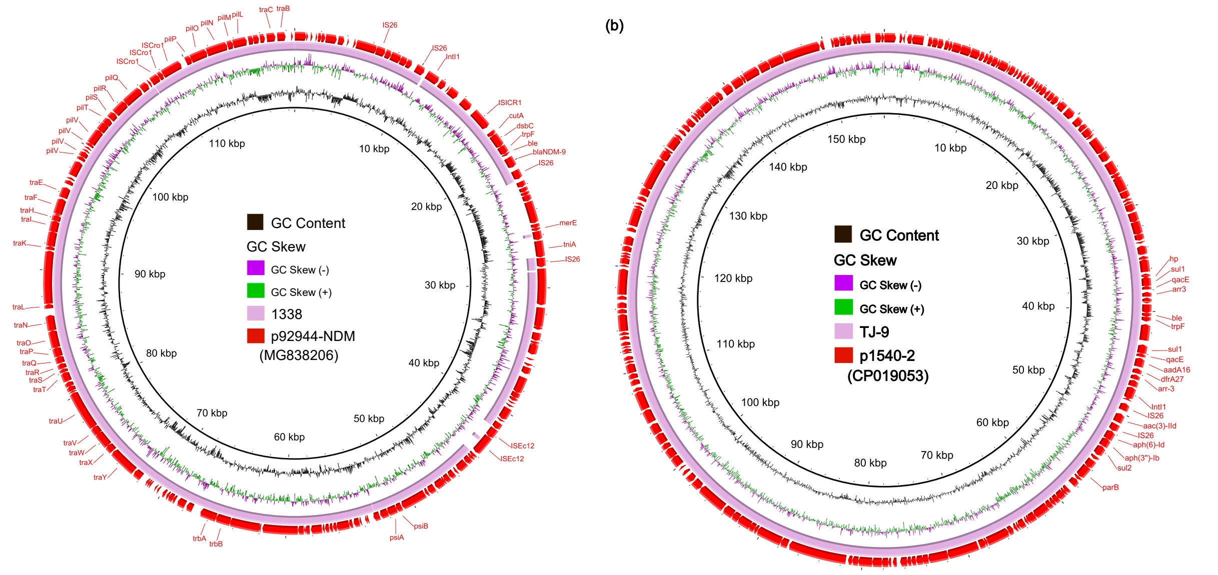

（a）Food-derived strain 1338 was found to contain p92944-NDM like plasmid, which belonged to IncB/O/K/Z type (the outmost circle). (b) Food-borne strains 1138 and 1249 were found to harbor pJH51-1-like plasmid, key genetic loci were labeled. Plasmid sequences in this study were generated from the Illumina sequencing data.

The type (b) genomic sequences involved the contigs of 118 isolates, including those recovered from clinical samples (n=46), pig feces collected from farm (n=29) and pork products purchased from supermarkets (n=43). The core genetic structure of the type (b) contigs was bla_{NDM}-*ble-trpF-dsbC-cutA,* surrounded by several IS sequences including IS*5*, IS*Aba25*, IS*3100* and/or TnAs3. Since IS*Aba25* is often truncated by IS*5* and associated with different insertion sites and deletion of some fragments, a variety of formats of △IS*Aba25*-IS*5*-△IS*Aba25* can be observed among these contigs. Two types of complete plasmids were directly acquired by 25 *E. coli* isolates, 14 of which had a size of ~42kb to ~46kb and exhibited a higher degree of sequence homology (100% similarity, 99.9% coverage) with an IncX3 type plasmid, namely pHNAH507-1 (Accession number: MH286945), which was collected from chicken fecal sample in a slaughterhouse in Anhui Province, China. The size of the remaining plasmids ranged from ~48kb to ~66kb, and shared >99.9% nucleotide sequence identity with the corresponding region of a 54,036 bp IncX3 plasmid, p362713-NDM (Accession number: MH909347), which was recovered from a *K. pneumoniae* strain in China. In addition to the difference in the size of the structural elements in △IS*Aba25*-IS*5*-△IS*Aba25* as described above, they also harbor two additional genetic structures, such as TnAs3-*groEL-groES-hp,* and IS*26*-*yjb*J-*yjbL*-$bla_{\mathrm{SHV\text{-}12}}$- IS*26* **(Fig S8)**.

Supplementary Figure S8. Structure alignment of two prevalent IncX3 type plasmids pHNAH507-1 and p362713-NDM.

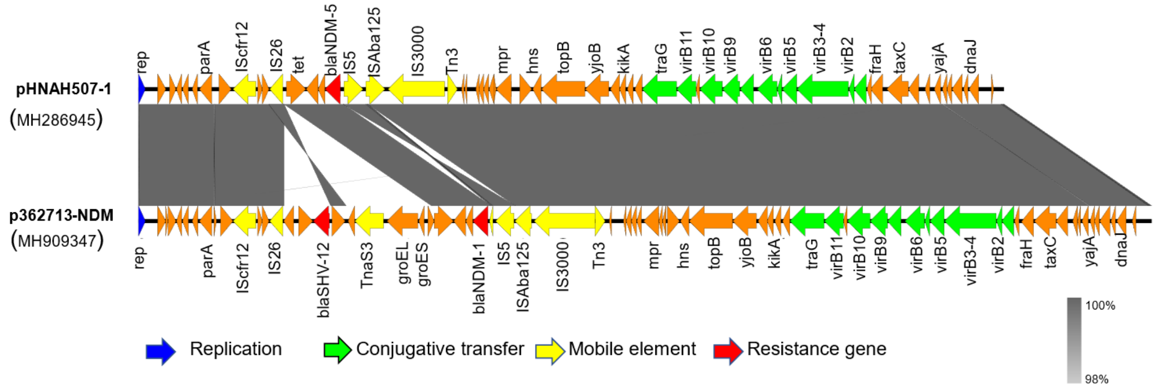

Among them, horizontal arrows depict the location, size, and orientation of predicted coding sequences with different functions by specified colors; red, carbapenemase determinant *bla*$_{NDM}$ and *bla*$_{SHV-12}$; yellow, genes that encode mobile elements; green, genes that encode the transfer protein (*tra*, and *vir* locus); blue, depicts replication genetic element, and a scale indicating the degree of similarity was shown at bottom right.

Supplementary Figure S9. Presence of IncX3 plasmid in E. coli strains of various ST types.

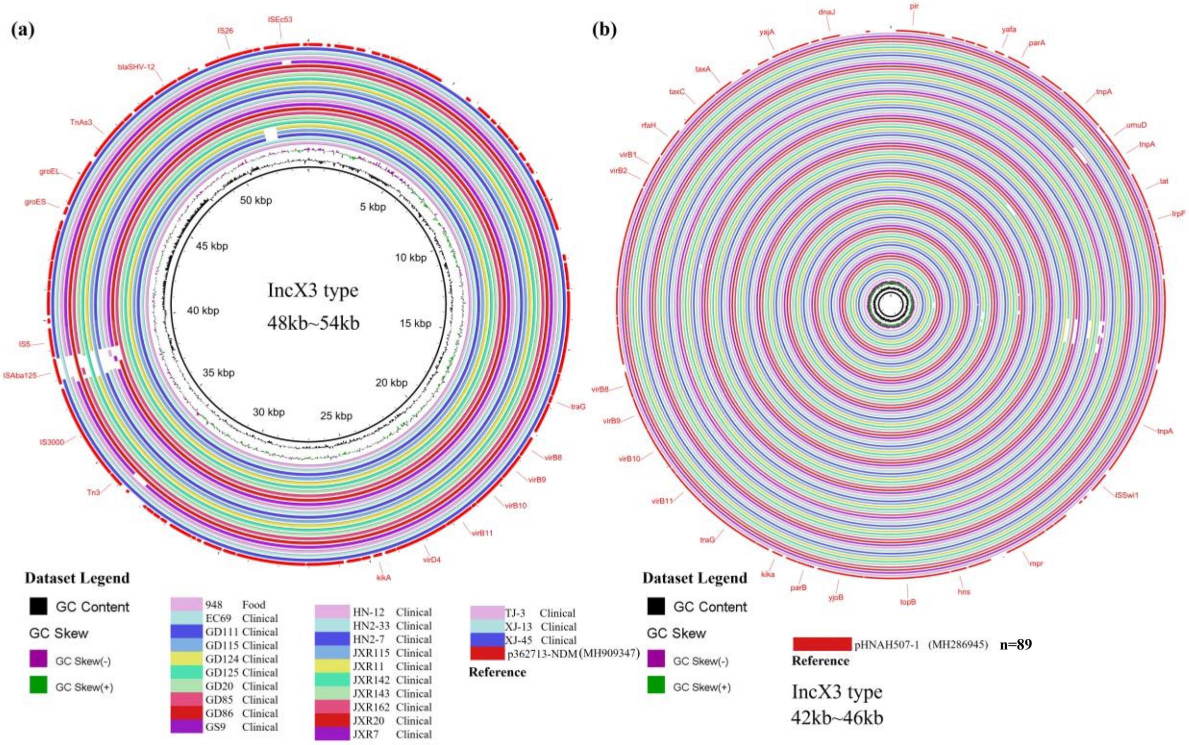

(a) Alignment of two IncX3 type plasmids p362713-NDM and pHNAHP507-1(NCBI database accession number MH9009347 and MH286945) with its best BLAST hit using the BLAST Ring Image Generator (BRIG). The sequence of (a) p362713-NDM and (b) pHNAHP507-1 in the outermost circle was used as the reference to determine the distribution pattern of the plasmids in this study; key genetic loci were labeled. Plasmid sequences were generated from the Illumina sequencing data.

The other 93 short contigs in type (b) fragments contained a truncated ΔIS*26* element at the initial 76bp, and the terminal sequences were partial mobile genetic elements such as ΔIS*5*, ΔIS*Aba125*, or IS*Swi1*, which were normally found to flank the core genetic structure of bla_{NDM} determinants. Among them, 85 contigs could align well with the two types of IncX3 plasmids as described above, 71 of which exhibited high homology to the IncX3 plasmid pHNAH507-1(~46kb), whereas the other 14 can be aligned well with plasmid p362713-NDM (~54kb) **(Fig S9)**. Importantly, this type of plasmid was mainly derived from clinical isolates, with a detection rate of 96% (24/25). Only one non-clinical strain, namely 948, which was collected from a food sample, was found to harbor this plasmid. It should be noted that this strain has a high probability of being assigned to a clinical isolate through the DAPC analysis. Furthermore, this type of IncX3 plasmids was rare in NCBI database, with only 25 such plasmids being recorded to date. These 25 plasmids were recovered from various pathogens including *Klebsiella pneumoniae* (n=10), *Enterobacter hormaechei* (n=2), *Escherichia coli* (n=4), *Enterobacter cloacae* (n=5) and *Citrobacter freundii* (n=4). Among them, 11 such plasmids were known to have originated from clinical strains isolated from urine, sputum, blood, and wound tissue samples. Besides, the plasmid screening results showed that the corresponding plasmid of the remaining contigs in type (b) fragments exhibited an extremely high level of sequence diversity. A total of four different plasmids of two plasmid replicon types (IncFII (n=5) and IncFII/IncFIA (n=1)), p974-NDM (**Fig S10a**) (Accession no. MG825370), pGZ3_NDM5 (**Fig S10b**) (Accession no. CP017981), pMC-NDM (**Fig S11**) (Accession no. HG003695) and pNDM5_020007 (**Fig S12**) (Accession no. CP025626) **(Table 2),** were recovered from six strains originated from food and clinical samples. The two remaining strains, with contig sizes of 1,407bp and 3,725bp respectively, did not exhibit any matches to plasmids in the GenBank database. Type (c) contigs were all collected from animal-borne strains (n=4). Complete contigs were 7464bp in length, comprised the predicted coding sequences and exhibited a GC content of 55.8%. The core genetic structure of bla_{NDM} elements in type (a) and (b) fragments

were similar, yet the flanking mobile elements IS*3100* were replaced by the *fepE* gene, which encodes an inner membrane transport protein. Analysis of the backbone of this *bla*NDM-bearing plasmid showed that it also belonged to IncX3. Further studies are required to confirm the function of this recombinant plasmid.

Supplementary Figure S10. Alignment of IncF plasmids p974-NDM and pGZ3_NDM5 (NCBI database accession number MG825370 and CP017981, respectively) with its best BLAST hit in this study.

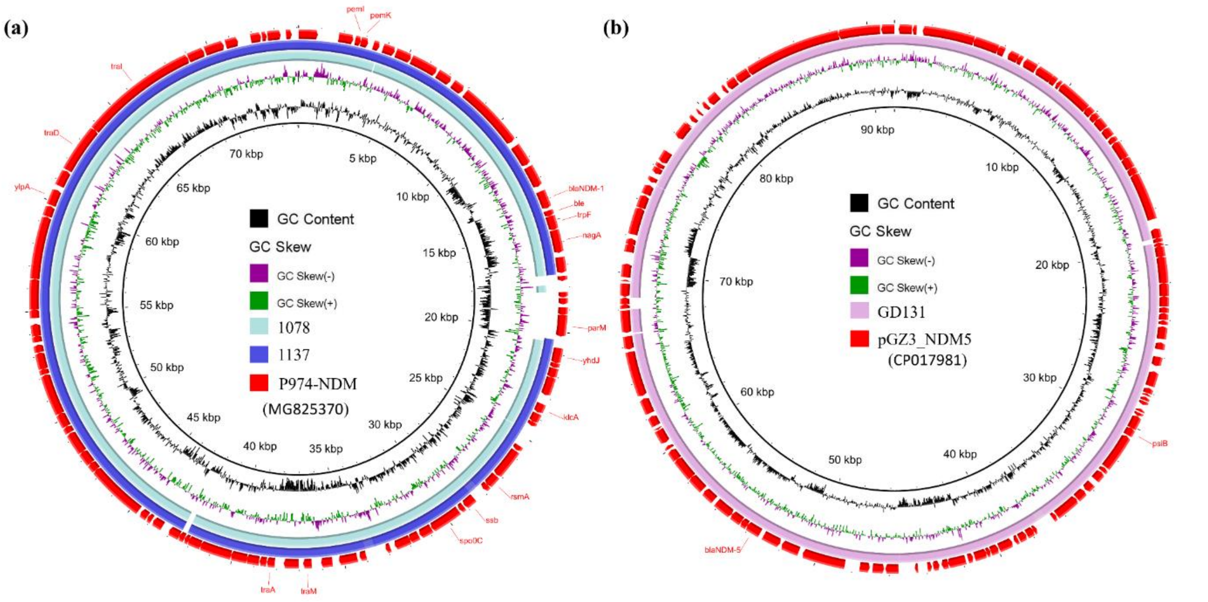

(a)Strains 1078 and 1137, 974 which belonged to ST10, ST 641 and ST5909, were found to contain p974-NDM-like plasmid using the BLAST Ring Image Generator (BRIG). p974-NDM plasmid was used as a reference (the outmost circle). (b) key genetic loci are labeled. Plasmid sequences in this study were generated from the Illumina sequencing data.

Supplementary Figure S11. Alignment of two plasmids pMC-NDM (NCBI database accession number HG003695) with its best BLAST hit in this study.

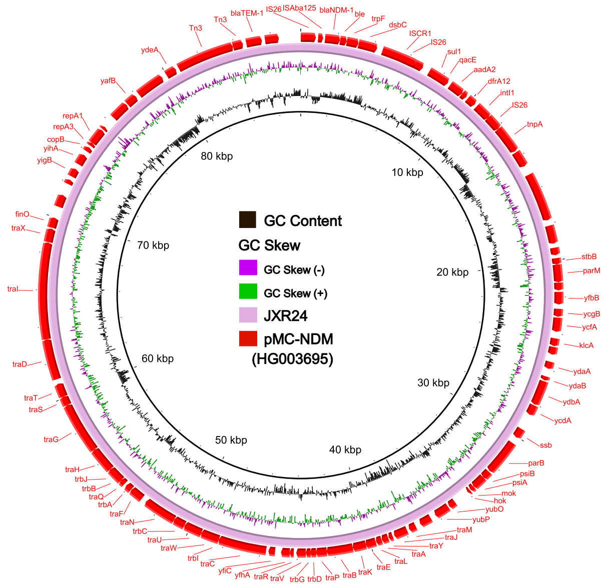

(a) Clinical strain JXR24 was found to harbor pMC-NDM-like plasmid, which belonged to IncFII type (the outmost circle), key genetic loci were labeled. Plasmid sequences in this study were generated from the Illumina sequencing data.

Supplementary Figure S12. Alignment of pNDM5_020007 (NCBI database accession number CP025626) with its best BLAST hit in this study.

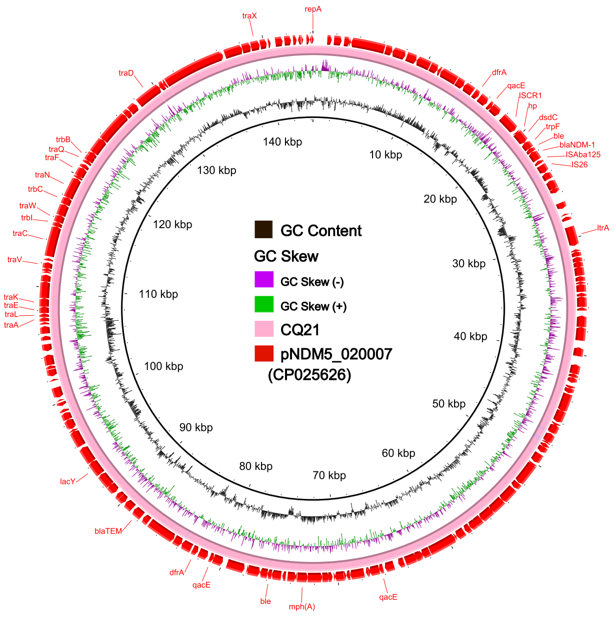

Clinical bacteria CQ21 was found to contain pLA-64-like plasmid using the BLAST Ring Image Generator (BRIG). The pNDM5_020007 plasmid was used as reference (the outmost circle); key genetic loci were labeled. Plasmid sequences in this study were generated from the Illumina sequencing data.

FIGURE S13. Transmission and evolution routes of *bla*NDM-bearing Escherichia coli in the ecosystem.

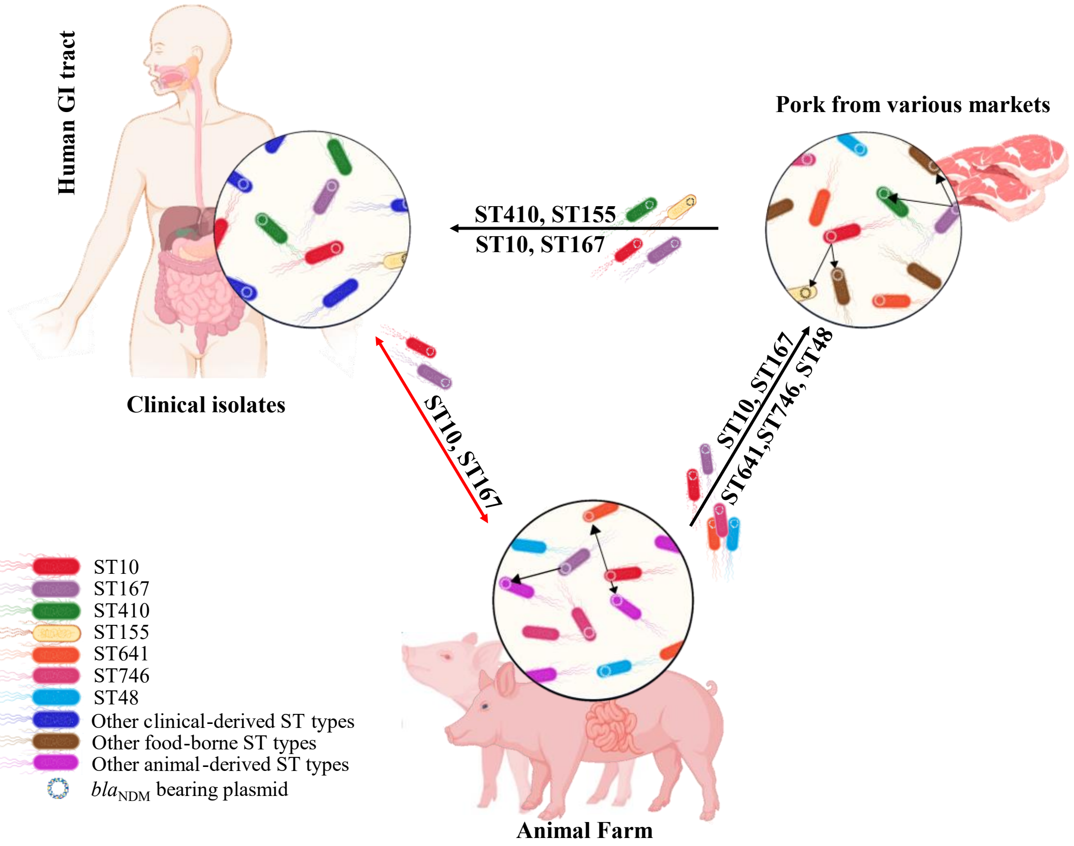

Possible transmission of STs of *bla*NDM-bearing E. coli strains among human, animals and pork. Transmission chains are represented by arrows by arrows in red and black colors. The circles illustrate the potential evolution model of *bla*NDM-bearing Escherichia coli in different hosts. Black arrows indicate the transmission events of *bla*NDM-bearing plasmids from common STs of strains to representative STs of strains in animal GI tract and food chains.
